# Emotional understanding in Japanese children 3–6 years: Language ability mediates the relations between theory of mind and executive function

**DOI:** 10.1371/journal.pone.0346175

**Published:** 2026-07-02

**Authors:** Awoun Jung, Izumi Uehara

**Affiliations:** 1 Department of Psychology, Ochanomizu University, Tokyo, Japan; 2 Institute for Education and Human Development, Ochanomizu University, Tokyo, Japan; Father Muller Charitable Institutions, INDIA

## Abstract

Previous research has documented associations between children’s emotional understanding (EU) and social-cognitive abilities such as theory of mind (ToM) and executive function (EF); however, findings in preschool-aged children remain inconsistent. One potential explanation is that language ability, which develops rapidly during early childhood, has often not been examined concurrently with these abilities. The present study focused on individual differences in EU and examined how ToM, EF, and language ability relate to EU in Japanese preschool children, with particular attention to the mediating role of language. Participants were 90 children aged 3–6 years (M = 60.34 months, SD = 10.82). Children completed a false belief task, emotional understanding tasks, executive function tasks assessing cognitive flexibility and working memory, and a receptive vocabulary test. Correlational analyses, hierarchical multiple regression analyses, and bootstrapped mediation analyses were conducted. EU was positively associated with ToM, EF, and language ability. Language ability accounted for significant variance in EU beyond age and gender. When language ability was included in the model, the association between ToM and EU was no longer significant, whereas the association between EF and EU remained significant but was attenuated. Mediation analyses revealed significant indirect effects of both ToM and EF on EU through language ability, indicating that the association between ToM and EU was accounted for by language ability, whereas EF showed both direct and indirect associations with EU. These findings suggest that language ability plays a central role in linking social-cognitive abilities to emotional understanding in early childhood. Theory of mind was associated with emotional understanding primarily through language, whereas executive function showed both direct and indirect associations via language ability. By modeling multiple predictors simultaneously, this study provides an integrated account of individual differences in emotional understanding and elucidates the distinct pathways through which cognitive and social-cognitive skills are associated with emotional development.

## Introduction

The development of social-cognitive abilities, including emotional understanding (EU) and theory of mind (ToM), is fundamental to young children’s socialization and interpersonal functioning [1,2]. Although ToM has long been a central focus of developmental research, EU has received less attention but has recently been recognized as an important construct warranting systematic investigation. EU refers to children’s developing capacity to identify, differentiate, explain, and predict emotional states in themselves and others [3]. Previous studies have shown that EU develops with age [4], with older children relying more on contextual cues when interpreting others’ emotions [5]. Despite this relatively robust developmental sequence, substantial individual differences in EU have been observed among children of the same age [4]. Some children exhibit relatively advanced understanding of emotional causes, consequences, and regulation, whereas others show more limited emotion knowledge despite comparable chronological age. Previous research suggests that such variability may arise from differences in children’s language development, social-cognitive abilities, family environments, and opportunities for emotion-related social interactions [6–8]. Identifying the factors underlying such differences is therefore a key issue in understanding early EU development. Accordingly, the present study focuses on individual differences in EU and examines the contribution of several cognitive and social-cognitive factors, including language ability.

ToM and EU are closely related but conceptually distinct aspects of social-cognitive development. ToM refers to the ability to understand that oneself and others possess mental states, such as beliefs, desires, intentions, and knowledge, that guide behavior [9]. In contrast, EU focuses specifically on the recognition, interpretation, explanation, and prediction of emotional states [4]. Whereas ToM concerns the broader understanding of mental representations, EU concerns knowledge about emotions and their causes, consequences, and regulation. Because emotions are closely linked to beliefs and desires, the two abilities are intertwined during development and are likely to influence one another throughout early childhood. A growing body of research has demonstrated significant associations between EU and ToM in preschool children, even after controlling for age [6,8,10], as well as in toddlers aged 2–3 years [11,12]. Consistent with these findings, Japanese studies have reported that children with more advanced EU and ToM engage in more successful social interactions [13,14]. Notably, Mizokawa and Koyasu [14] suggested that understanding hidden emotions may not facilitate social interaction when ToM is insufficiently developed. Longitudinal studies have further proposed that ToM serves as a foundation for EU development. For example, de Rosnay et al. [8] argued that ToM provides a cognitive basis for later emotional understanding. Hughes and Dunn [15] showed that ToM at age 4 predicted children’s understanding of negative emotions at age 7. Similarly, Seidenfeld et al. [16] found that ToM significantly predicted emotion knowledge one year later in children aged 3–5 years.

However, evidence also points to the opposite developmental direction. Eggum et al. [17] reported that EU at 3 years of age predicted ToM at 4 and 6 years, whereas ToM at 4 years predicted EU at 6 years. Similarly, O’Brien et al. [18] found that emotion knowledge at 3 years predicted ToM at 4 years, even after controlling gender, socioeconomic status, and receptive language. Using a large longitudinal sample of children aged 3–8 years, Grazzani et al. [7] further demonstrated that EU predicts ToM across ages and genders, with language ability mediating this association. Taken together, these findings suggest a bidirectional and developmentally dynamic relationship between EU and ToM. Nevertheless, the mechanisms underlying this reciprocal association, as well as the conditions under which one ability may precede the other, remain unclear.

Language ability has long been regarded as a critical factor in children’s social and social–cognitive development, including EU and ToM [19,20]. Children’s verbal skills are significantly associated with both executive function [21] and emotional competencies [7,8]. Conte et al. [22] reported that emotion knowledge and language ability in children aged 24–47 months were positively associated with ToM, even after controlling for age and gender. Other studies have highlighted reciprocal relations between language and socio-emotional development. For example, Strand et al. [23], reported a bidirectional association between EU and receptive language ability in children aged 49–67 months. Moreover, ToM at 4 years has been shown to correlate with language ability at both 2 and 4 years [24], and similar associations have been observed in children under 7 years [25]. Several studies further suggest that language ability predicts subsequent ToM acquisition [26,27]. Macheta et al. [28] demonstrated that language ability may serve a scaffolding role in the development of emotional and cognitive state understanding. However, it remains unclear whether language functions as a foundational mechanism that organizes and integrates social-cognitive representations or reflects a correlated developmental outcome.

Executive function (EF) is another cognitive domain that may contribute to individual differences in EU. EF refers to higher-order cognitive processes, including working memory, cognitive flexibility, and inhibitory control, and supports emotional regulation, social competence, and academic achievement. Several studies have reported associations between EF and emotional competence [29,30], whereas others suggest that early emotional regulation predicts later EF development [31,32]. EF and ToM are thought to share overlapping neural substrates in the frontal lobe and develop rapidly during early childhood. Accordingly, EF may facilitate children’s ability to integrate contextual, linguistic, and mental-state information when interpreting others’ emotions. Prior research has also suggested that EF plays a role in understanding false or concealed emotions [33,34].

EF is typically conceptualized as comprising working memory, cognitive flexibility, and inhibitory control [35], with working memory and cognitive flexibility particularly implicated in emotional processing [36,37]. Japanese studies have likewise reported associations between EF components and EU, with set shifting and working memory predicting performance on context-based emotional comprehension tasks [38]. Nevertheless, empirical findings regarding the EF–EU association remain limited and somewhat inconsistent. Furthermore, although previous studies have examined the associations among EU, ToM, EF, and language ability, these relationships have often been investigated separately. For example, some studies have focused on the mediating role of language in the association between ToM and EU [7], whereas others have examined language as a mediator linking EF and emotional development [39,40]. Consequently, it remains unclear whether ToM and EF make unique contributions to EU and whether language ability serves as a common mechanism underlying both pathways. Few studies have simultaneously examined these four abilities within a single analytic framework.

EU, ToM, EF, and language skills developed rapidly during the preschool years; however, few studies have examined the interrelationships among these four domains within a single framework. Clarifying how these abilities are interrelated is essential for understanding the mechanisms underlying individual differences in EU development. Accordingly, the present study investigated the associations among EU, ToM, EF, and language ability in young children. Given the limited attentional and motivational capacities of preschoolers, we selected a restricted set of well-established tasks to assess each construct. EU was measured using a widely used emotion-labeling task in Japan, together with Component VII of the Japanese version of the Test of Emotion Comprehension (TEC), which has been developed and partially validated by Japanese researchers with permission from the original authors. Because there is no consensus regarding the optimal assessment of ToM, we employed the false belief task, which has long been regarded as a standard and representative measure of ToM. This choice was also consistent with our aim of examining EU in relation to children’s general understanding of others’ mental states. Thus, in the present study, ToM refers specifically to mental-state understanding as indexed by false belief performance. EF was assessed using the Dimensional Change Card Sort (DCCS) and working memory tasks. Inhibitory control tasks were not included, as both our preliminary analyses and prior Japanese research [38] have indicated that inhibitory control is a less sensitive index of EF in relation to EU among Japanese preschoolers. Language ability was measured using the PVT-R, a receptive vocabulary test that is currently the most widely accepted measure of language ability for this age group in Japan.

By integrating EU, ToM, EF, and language ability within a single analytic framework, the present study sought to clarify how these abilities jointly contribute to individual differences in emotional understanding during early childhood. In particular, we examined whether language ability mediates the associations of ToM and EF with EU, thereby providing a more comprehensive account of emotional understanding than approaches that consider these abilities separately. We hypothesized that preschoolers’ EU would be positively associated with ToM, EF, and language ability. We further expected that ToM, EF, and receptive language ability would each uniquely contribute to individual differences in EU, given the substantial variability in EU observed across early childhood [4]. Finally, based on prior findings [7,39,40], we hypothesized that receptive language ability would mediate the associations of ToM and EF with EU.

## Methods

### Participants

The sample comprised 90 typically developing children (46 girls) with a mean age of 60.34 months (SD = 10.82; range = 42–80 months). Participants were recruited from kindergartens and nursery schools in Japan between October 2019 and February 2022 and were distributed across different preschool grades. All children were native Japanese speakers. Most participants came from middle-class families. Mothers’ educational attainment ranged from completion of middle school to a graduate degree. Maternal educational attainment was as follows: middle school (1.1%), high school (15.6%), some college (30%), bachelor’s degree (45.6%), and master’s or doctoral degree (6.7%). One participant did not report maternal educational attainment. Parental educational attainment was not used as a recruitment criterion and reflects the characteristics of the participating families.

### Ethics statement

The study was conducted in accordance with the guidelines of the Ethics Committee of Ochanomizu University (approval number: 2018-48). Written informed consent was obtained from all parents, and all procedures conformed to the Declaration of Helsinki.

### Measures

Children were tested individually in a quiet room at their kindergarten or nursery school. Standardized instruments assessing the variables of interest were administered during a single assessment session. The PVT-R was administered first as an introductory task. Following its completion, children were given a five-minute break. The remaining tasks were then administered in a counterbalanced order. Additional five-minute breaks were provided as needed depending on each child’s attention and fatigue level, resulting in a total of one to three rest periods during the assessment session. During breaks, children were allowed to rest and engage in brief informal conversation with the examiner. All measures were validated and appropriate for preschool-aged children.

#### Emotional understanding.

Emotional understanding (EU) was assessed using two tasks: an emotion-labeling task [41] and Component VII of the Test of Emotion Comprehension [TEC-VII [Hiding]; 42]. For the emotion-labeling task, short scenarios designed to elicit five emotions (happiness, sadness, anger, surprise, and fear) were created based on previous studies [43,44]. To confirm that the scenarios elicited the intended emotions, a pilot study was conducted with 10 adults. Based on the results, minor wording adjustments were made to ensure comprehensibility for young children. The protagonist was named “Taro” for boys and “Hanako” for girls. An example scenario (happiness), read aloud by the experimenter, was: “Today is Taro’s birthday. Taro’s friend brought him a birthday gift. How do you think he feels?” Participants received 1 point if they failed to identify the character’s emotion, 2 points if they correctly judged the emotion as positive or negative without specifying it, and 3 points if they correctly identified the specific emotion.

The second task was the Japanese version of TEC-VII, developed by Mizokawa and Koyasu [45]. This task consists of a picture book depicting four brief scenarios (two positive and two negative), each accompanied by four facial expressions representing different emotional outcomes. The examiner read a short description (e.g., “Haruo is showing his new bike to Susumu. Because Susumu does not have a bike, Haruo hides his true feelings. What is Haruo really feeling?”), after which the child selected the facial expression that best matched the character’s emotion. One point was awarded for each correct response, yielding a maximum score of 4. Scores for the two tasks were summed to create a composite EU score (maximum = 19).

#### Theory of mind.

Theory of mind (ToM) was assessed using two first-order false belief tasks: the Sally–Anne task [46] and the Smarties task [47].

In the Sally–Anne test, children were asked to predict where a character (Sally) would look for an object (bread) that had been moved to a new location without her knowledge. The story was presented in five illustrated panels. The experimenter asked a control question (“Where is the bread now?”) followed by a belief question (“Where will Sally look for her bread?”). One point was awarded only if both questions were answered correctly. In the Smarties task, children were first asked to guess the contents of a box that appeared to contain sweets. After responding, they were shown that the box actually contained a miniature duck. They were then asked what another person, who had not seen the contents of the box, would think was inside it. The experimenter asked a control question (“What is in the box?”) and a belief question (“What will the child’s mother, who has not seen inside the box, think is in it?”). One point was awarded only if both responses were correct. Scores from the two tasks were summed to yield a total ToM score, with a maximum of 2.

#### Executive function.

Executive function (EF) was assessed using two tasks: the Dimensional Change Card Sort [DCCS; 48] and a reverse text task [49]. The DCCS was used to assess cognitive flexibility and involved two dimensions (color and shape) administered in pre- and post-switch phases. Children were presented with two target cards (a red flower and a blue car) and a set of test cards (red cars and blue flowers) that differed from the targets on both dimensions. In the pre-switch phase, children sorted the cards by color; in the post-switch phase, they sorted the cards by shape. Children typically completed all six pre-switch trials correctly; therefore, children who failed this phase were assigned a score of 0 and excluded from post-switch analyses. Only post-switch performance was analyzed, with scores ranging from 0 to 6.

Working memory was assessed using a reverse text task [50,51]. The experimenter read aloud a sequence of numbered words, after which children were asked to indicate the corresponding numbers on a sheet displaying the words in reverse order. Following an example and a practice trial, children completed the task, which consisted of two trials each for two-, three-, four-, and five-word sequences. Children advanced to the next sequence length if they responded correctly on at least one of the two trials. All words were age-appropriate for children aged 3 years and older. Scores ranged from 1 to 5. Scores from the DCCS and reverse text tasks were summed to yield a composite EF score, with a maximum of 11.

#### Picture Vocabulary Test – Revised.

Children’s receptive vocabulary was assessed using the standardized Japanese version of the Picture Vocabulary Test–Revised [PVT-R; 52]. The PVT-R is designed for children aged 3–12 years and consists of four numbered illustrations, from which the child selects the picture corresponding to a word spoken by the examiner. Scoring followed standard procedures.

### Data analysis

Descriptive statistics were first computed for all variables, followed by correlation analyses. Hierarchical multiple regression analyses were then conducted to examine the extent to which ToM, EF, and language ability predicted EU. Finally, the mediating role of language ability in the associations between ToM and EF and EU was tested. Indirect effects were evaluated using bias-corrected bootstrapping with 2,000 resamples, implemented in AMOS (version 28.0; IBM Corp., Armonk, NY, USA). Mediation was considered significant when the 95% confidence interval did not include zero.

### Use of large language models

Large language models were used solely to support English paraphrasing and grammatical refinement of the manuscript. Specifically, ChatGPT (a large language model developed by OpenAI) was employed to improve clarity, consistency, and linguistic precision. The model was not used for data analysis, hypothesis generation, interpretation of results, or decision-making regarding study design.

## Results

### Descriptive statistics and correlations between variables

Descriptive statistics and bivariate correlations among the study variables are presented in Table 1. Age was significantly correlated with receptive vocabulary (PVT-R) and EU. In addition, EU showed significant positive correlations with ToM (*r* = .29, *p* < .01) and EF (*r* = .38, *p* < .001). Receptive vocabulary was also significantly associated with EU (*r* = .55, *p* < .001). These results indicate that EU is related to multiple cognitive and social–cognitive abilities during the preschool period.

**Table 1:** Descriptive statistics and results of correlation analyses.

	Mean	SD	1	2	3	4	5	6
1. Age (m)	60.34	10.82	—					
2. Gender	—	—	.24*	—				
3. PVT-R score	20.32	10.44	.54***	.05	—			
4. EU score	13.21	2.58	.47***	.25*	.55***	—		
5. ToM score	1	0.75	.37***	.18	.36***	.29**	—	
6. EF score	8.01	1.98	.30**	.15	.36***	.38***	.21	—

EF, executive function; EU, emotional understanding; PVT-R, Picture Vocabulary Test-Revised; SD, standard deviation; ToM, theory of mind.

* *p* < 0.05, ** *p* < 0.01, *** *p* < 0.001.

### Factors predicting emotional understanding

To examine whether ToM and EF explained variance in EU, a hierarchical multiple regression analysis was conducted using AMOS (Version 28.0), with age and gender included as covariates.

As shown in Table 2, age and gender were entered in Step 1. The model was significant, *F* (2, 87) = 13.67, *p* < .001, accounting for 22% of the variance at EU. Of these variables, only age was a significant predictor (*β* = .44, *p* < .001). At Step 2, ToM and EF were added, resulting in a significant increase in explained variance (*ΔR*² = .05, *p* < .001), with the total R^2^ increasing to.27. EF emerged as a significant predictor of EU (*β* = .24, *p* < .05). At Step 3, receptive vocabulary was entered, accounting for an additional 9% of the variance (*ΔR*² = .09, *p* < .001), with the total R² reaching.36. The final model was significant, *F* (5, 84) = 10.85, *p* < .001. Receptive vocabulary was a strong predictor of EU (*β* = .38, *p* < .001), whereas age and EF were no longer significant. These findings suggest that language ability plays a central role in explaining individual differences in EU and may account for the association between EF and EU.

**Table 2:** Ability of PVT-R, theory of mind, and executive function scores, and age and gender, to predict emotional understanding according to hierarchical regression analysis.

	*β*	SE	*t*	*p*
**Step 1**				
Age (m)	.436***	0.023	4.526	0.000
Gender	.142	0.494	1.478	0.143
**Step 2**				
Age (m)	.335**	0.024	3.273	0.002
Gender	.112	0.483	1.196	0.235
ToM score	.096	0.340	0.975	0.333
EF score	.239*	0.125	2.490	0.015
**Step 3**				
Age (m)	.169	0.025	1.580	0.118
Gender	.157†	0.458	1.763	0.081
ToM score	.032	0.325	0.340	0.735
EF score	.159†	0.121	1.715	0.090
PVT-R score	.377**	0.026	3.531	0.001

EF, executive function; EU, emotional understanding; PVT-R, Picture Vocabulary Test-Revised; SE, standard error; ToM, theory of mind.

† *p* < 0.10, * *p* < 0.05, ** *p* < 0.01, *** *p* < 0.001.

### Mediation models

Mediation analyses were conducted to examine whether language ability mediated the associations of ToM and EF with EU. Based on the preceding analyses, language ability was specified as a mediator linking ToM and EF to EU. Gender was excluded from the mediation model because it did not account for significant variance in EU in the hierarchical regression analysis and was not significantly correlated with ToM or EF. Age was also excluded because, although it was correlated with language ability, it was not a significant predictor of EU in the final regression model. All mediation analyses were conducted using AMOS (Version 28.0).

**Fig 1:**
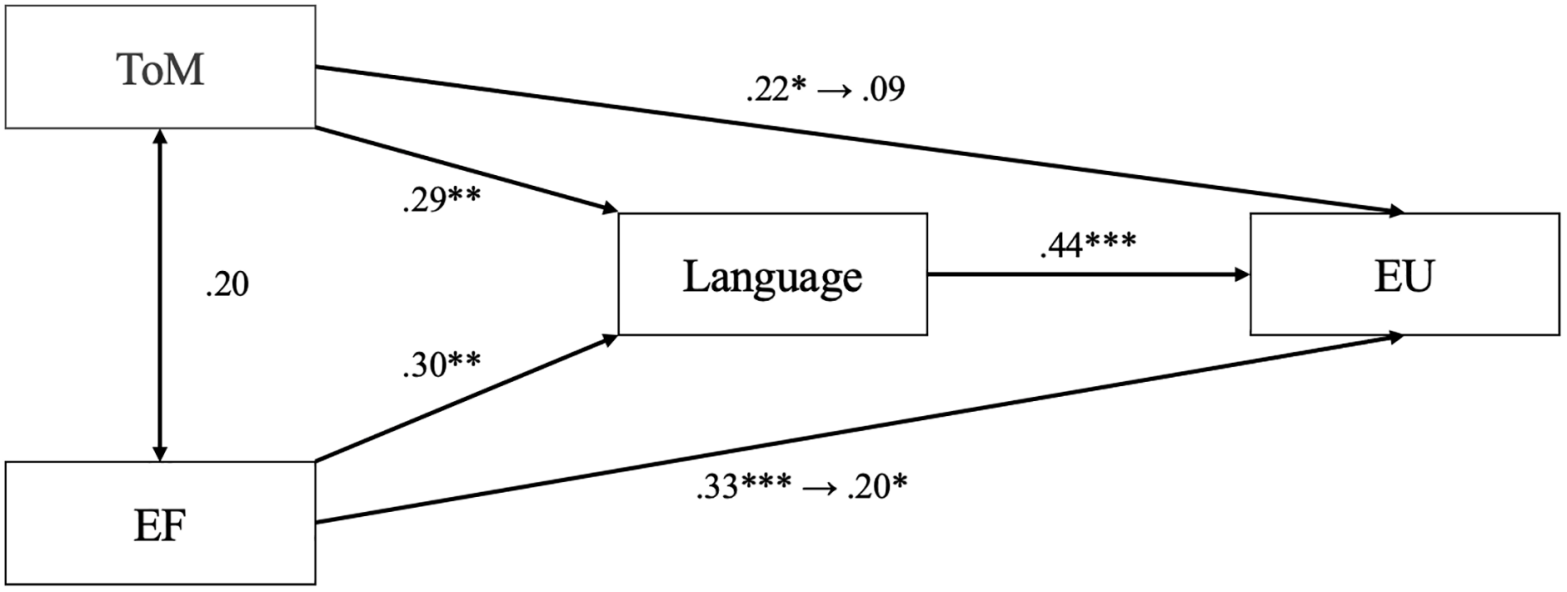
Language ability as a mediator of the relationships between ToM and EF and EU. EF, executive function; EU, emotional understanding; Language, Picture Vocabulary Test – Revised score; ToM, theory of mind. Demographic covariates are not shown. *p < 0.05. **p < 0.01. ***p < 0.001.

As shown in Fig 1, ToM and EF exhibited significant standardized direct effects on EU (*β* = .22, *p* < .05 and *β* = .33, *p* < .001, respectively). However, after language ability was included as a mediator, the direct effect of ToM on EU was no longer significant (*β* = .09, *p* = .31), whereas the direct effect of EF on EU was attenuated but remained statistically significant (*β* = .20, *p* = .03). ToM and EF were both significantly associated with language ability (*β* = .29, *p* < .01; *β* = .30, *p* < .01), which in turn was strongly associated with EU (*β* = .44, *p* < .001).

Model fit indices indicated acceptable, though not optimal, fit. The CFI suggested adequate fit (CFI = .93), whereas the TLI fell below conventional criteria (TLI = .79). The chi-square test was marginally significant, *χ*² (2) = 5.87, *p* = .05. Accordingly, the model fit should be interpreted with caution. Despite these limitations, indirect effects were evaluated using bias-corrected bootstrap confidence intervals, which are robust to violations of normality. Bootstrap analyses indicated significant indirect effects for the paths from ToM to EU (95% CI = [.04,.24]) and from EF to EU (95% CI = [.05,.24]), via language ability, as neither interval included zero. Overall, the mediation model accounted for 31.8% of the variance in EU.

## Discussion

The present study examined the relationships among emotional understanding (EU), theory of mind (ToM), executive function (EF), and language ability in Japanese preschool children aged 3–6 years. Previous research has documented close associations between emotional understanding and social-cognitive abilities, yet findings have been mixed, particularly in early childhood. One possible explanation is that language ability—an essential developmental resource during the preschool years—has often been examined separately or insufficiently integrated into models of emotional and social-cognitive development. To address this gap, the present study simultaneously examined ToM, EF, and language ability and explored how these abilities jointly relate to children’s emotional understanding.

Consistent with previous research, emotional understanding was positively associated with both theory of mind and executive function. These findings replicate earlier evidence that children with more advanced mental state reasoning tend to show greater sensitivity to others’ emotional states [e.g., 5,53,54], and that executive function is linked to emotional understanding through its role in attention control, cognitive flexibility, and behavioral regulation [34,38]. Importantly, the present study extends this literature by demonstrating these associations in a Japanese preschool sample, a population that has received relatively limited empirical attention in research on emotional development.

However, a different pattern emerged when language ability was included in the model. The direct effect of theory of mind on emotional understanding was no longer significant, suggesting that this association was largely accounted for by language ability. In contrast, executive function remained a significant predictor of emotional understanding, although its effect size was reduced. These findings indicate that language ability partially mediates the association between executive function and emotional understanding and appears to account for the association between theory of mind and emotional understanding. Mediation analyses further supported this interpretation, revealing significant indirect effects of both theory of mind and executive function on emotional understanding through language ability. Together, these findings suggest that language may serve as a developmental pathway through which these abilities are expressed in emotion-related tasks.

The prominent mediating role of language ability observed in this study is theoretically meaningful. Emotional understanding requires children to identify, label, and differentiate emotional states, infer emotional causes, and integrate contextual and social information. These processes place substantial demands on receptive vocabulary and linguistic representation. Thus, while theory of mind and executive function provide important cognitive foundations, language ability appears to function as a critical interface through which social-cognitive abilities are translated into explicit emotion knowledge—particularly in the case of theory of mind and, to a lesser extent, executive function. Language may not merely reflect emotion knowledge but may actively structure children’s conceptual organization of emotional experiences.

This interpretation is consistent with previous findings emphasizing the central role of language in emotional development. For example, Grazzani et al. [7] reported that receptive vocabulary fully mediated the association between theory of mind and emotional understanding in children aged 3–8 years. Similarly, a longitudinal study by Wang et al. [40] demonstrated that early cognitive flexibility predicted later emotional understanding indirectly via language ability. Importantly, previous studies have typically examined the relations among ToM, EU, and language or among EF, EU, and language separately. By integrating these abilities within a single framework, the present study highlights language ability as a potentially central mechanism through which multiple cognitive and social-cognitive skills contribute to emotional understanding. This perspective extends previous work by suggesting that language is not simply one predictor among many but may play a pivotal role in connecting children’s broader cognitive capacities with their developing understanding of emotions.

Importantly, the differing patterns observed for ToM and EF provide further insight into the mechanisms underlying emotional understanding development. The association between theory of mind and emotional understanding was fully accounted for by language ability, whereas executive function remained a significant predictor of emotional understanding even after language ability was included in the model. This pattern suggests that language may be particularly important for translating children’s understanding of mental states into explicit emotion knowledge, while executive function may contribute to emotional understanding through both language-related and nonlinguistic cognitive processes.

More broadly, these findings suggest that language serves not merely as a correlate of emotional understanding but as an important developmental mechanism through which children organize and communicate emotional experiences. This interpretation is broadly consistent with Vygotskian perspectives that view language as a psychological tool supporting the development of higher mental functions [55,56].

In the present study, no significant association was observed between executive function and theory of mind. This finding is consistent with the meta-analysis by Devine and Hughes [57], which reported modest and variable associations between these abilities in early childhood. Although some studies suggest that executive function plays a critical role in the development of theory of mind [e.g., 33,34], the strength and direction of this relationship appear to vary across samples and developmental periods. This variability underscores the importance of modeling multiple predictors simultaneously, as developmental relations among cognitive systems may differ across cultural and methodological contexts.

One possible explanation for the absence of a significant association involves cultural differences in the developmental trajectory of theory of mind. Cross-cultural research has shown that children in Japan and Austria tend to acquire false belief understanding later than children in English-speaking countries [58,59]. Given the limited number of studies examining the relationship between executive function and theory of mind in Japanese children, further research is needed to determine whether cultural factors moderate the association between these abilities.

Although cultural differences may influence the developmental trajectory of theory of mind, the role of language in emotional development may be more broadly shared across cultural contexts. The present findings may be particularly meaningful within the context of Japanese preschool education and socialization. Japanese cultural practices often emphasize social harmony, situational sensitivity, and implicit understanding rather than explicit verbalization of internal states. In such environments, children may rely more heavily on language ability to make emotion knowledge explicit and accessible. From this perspective, the strong mediating role of language observed in the present study may reflect not only culturally shaped pathways but also broader developmental processes linking social cognition and emotional understanding. Similar associations among language ability, theory of mind, and emotional understanding have been reported in Western samples [7], suggesting that language may represent an important mechanism through which social-cognitive abilities support emotional understanding across diverse cultural settings. Accordingly, the present findings may generalize most readily to the role of language as a developmental mechanism rather than to the specific magnitude of the observed effects. Therefore, researchers and educators in other countries may likewise benefit from considering language ability when examining individual differences in emotional understanding and designing interventions to support socio-emotional development. However, this interpretation remains an empirical question, and future cross-cultural research is needed to determine whether the strength and nature of these associations vary across cultural contexts.

From a theoretical perspective, the present study supports a language-centered model of emotional development in early childhood. By integrating theory of mind, executive function, and language ability within a single analytic framework, the findings provide a more coherent account of how emotional understanding develops than models examining these abilities in isolation. Language ability appears to play a crucial role in organizing and linking social-cognitive processes with emotion knowledge.

From an applied perspective, the results highlight the importance of language-based interventions in early childhood education. Enhancing children’s receptive vocabulary and emotion-related language may support not only emotional understanding but also broader social-cognitive development. Educational practices that encourage rich emotional discourse may therefore be particularly effective in fostering children’s emotional and social competence.

From a clinical perspective, the findings suggest that assessments of emotional difficulties in preschool children may benefit from considering language ability, as limitations in emotional understanding may partly reflect differences in receptive vocabulary rather than emotional competence alone. The results further raise the possibility that interventions targeting language development may indirectly support children’s emotional understanding by facilitating the expression and application of broader social-cognitive abilities, including theory of mind and executive function.

Several limitations should be acknowledged. First, the cross-sectional design and relatively small sample size preclude causal inferences. Longitudinal studies with multiple assessment points are needed to clarify developmental pathways among emotional understanding, theory of mind, executive function, and language ability. In addition, participants were recruited primarily from middle-class families, and more than half of the mothers held a bachelor’s degree or higher. Consequently, the findings may not generalize fully to children from more socioeconomically and educationally diverse backgrounds. Second, the assessment of emotional understanding was limited to two components of emotion scripts, using labeling tasks and part of the Test of Emotion Comprehension. Similarly, theory of mind, executive function, and language ability were assessed with a limited set of tasks. Future research should employ a broader range of measures to examine whether task-specific differences influence the observed relationships.

Finally, family-related factors such as parental emotion talk, caregiving attitudes, socioeconomic status, and parental education were not examined in the present study. Previous research has shown that children’s emotional understanding is influenced by parents’ emotional expressiveness and responsiveness [60], the use of emotion-related language in parent–child interactions [8], and broader family background factors linked to language development [61]. Incorporating these variables into future models will provide a more comprehensive account of emotional development in early childhood.

## Conclusion

The present study demonstrates that theory of mind and executive function contribute to individual differences in emotional understanding during early childhood, with language ability serving as an important developmental pathway linking these abilities to emotional understanding. These findings highlight the importance of considering language alongside cognitive and social–cognitive abilities when examining emotional development in preschool children.

## Supporting information

S1 Dataset Anonymized dataset used in the present study. (CSV)
